# Acute ischemic stroke care in Germany – further progress from 2016 to 2019

**DOI:** 10.1186/s42466-021-00115-2

**Published:** 2021-04-01

**Authors:** Daniel Richter, Ralph Weber, Jens Eyding, Dirk Bartig, Björn Misselwitz, Armin Grau, Werner Hacke, Christos Krogias

**Affiliations:** 1 Department of Neurology, St. Josef-Hospital Bochum, Ruhr University Bochum, Gudrunstr. 56, 44791 Bochum, Germany; 2 Medical Faculty, Ruhr University of Bochum, Bochum, Germany; 3 Department of Neurology, Alfried Krupp Krankenhaus Essen, Essen, Germany; 4 Department of Neurology, Gemeinschaftskrankenhaus Herdecke, Herdecke, Germany; 5 Institute of Quality Assurance Hesse, Eschborn, Germany; 6 Department of Neurology, Klinikum der Stadt Ludwigshafen, Ludwigshafen, Germany; 7 Department of Neurology, University of Heidelberg, Heidelberg, Germany

**Keywords:** Stroke, Thrombolysis, Thrombectomy, Stroke unit care, Health care structure

## Abstract

**Background:**

Stroke Unit Care (SUC), intravenous thrombolysis (IVT) and mechanical thrombectomy (MT) are evidence-based treatment options for acute ischemic stroke (AIS). Using nationwide comprehensive administrative data from Germany, we recently reported nationwide development of AIS admissions, SUC rates, IVT rates and MT rates in Germany between 2010 and 2016. In this update paper, we analyze data on the further development of these data to 2019 after publication of time window extensions for recanalization therapies.

**Methods:**

We considered all hospitalized cases with the main diagnosis of the ICD-10-GM code I63 (AIS) for the year 2019. We identified stroke therapies by using the corresponding Operating and Procedure Keys for IVT, MT and SUC out of the DRG statistics. Regional analyses are based on data from the 412 German administrative districts and cities. We compared the results with those from 2016.

**Results:**

Number of hospitalized AIS patients showed a mild decrease in 2019 (*n* = 225,531) compared with 2016 (*n* = 227,687), with significant more AIS patients treated on a stroke unit in 2019 (*n* = 167,799; 74.4% vs. *n* = 164,270; 72.1%, *p* < 0.001). The rate of IVT further increased from 14.9% (*n* = 33,916) in 2016 to 16.3% (*n* = 36,745) in 2019 (*p* < 0.001). Similarly, the MT rate increased from 4.3% (*n* = 9795) in 2016 to 7.2% (*n* = 16,135) in 2019 (*p* < 0.001). There was still a high regional variability for MT (1.4 to 15.2%) according to the place of residence of the AIS patients.

**Conclusions:**

In Germany*,* the rates of recanalization therapies in patients with AIS continued to increase from 2016 to 2019. Compared to IVT-rates and numbers, the respective data for MT procedures showed an even more pronounced increase.

## Background

Inpatient stroke cases have increased over the last decade in Germany [1]. In 2019, we reported nationwide numbers of all hospitalized patients with acute ischemic stroke (AIS) in Germany for the years 2010 to 2016 and the distribution and evolution of recanalizing treatment strategies such as intravenous thrombolysis (IVT) and mechanical thrombectomy (MT) [2]. In 2015, MT had become an evidence-based and approved treatment strategy based upon five randomized trials showing the safety and efficacy of thrombectomy with stent retrievers in patients with large vessel occlusion (LVO) of the anterior circulation up to 12 h after onset of symptoms [3–7]. Therefore, MT has become the third evidence-based pillar of acute stroke therapy, in addition to stroke unit care (SUC) and IVT with recombinant tissue plasminogen activator (rt-PA) [8–11]. It has been estimated that 33 to 36% of all AIS patients could be candidates for IVT [12, 13], and 10 to 13% for MT [14, 15].

In the meantime, several randomized trials on MT and IVT have established these two treatment strategies also for selected patients presenting up to 24 h after symptom onset or with an unknown time window [16–20]. Furthermore, MT has been shown to be safe and effective in AIS patients with contraindication for IVT and in posterior circulation stroke [21, 22]. In Germany, the German Stroke Society (DSG) has also introduced neurovascular networks to improve multidisciplinary acute stroke care [23].

Based on administrative data from all acute care hospitals in all 412 cities/regions of Germany, we compare the nationwide evolution of IVT, MT and SUC in hospitalized AIS patients from 2019 with the previous data from 2016. Furthermore, our evaluation represents the most current data before the coronavirus disease 2019 (COVID-19) outbreak and can therefore serve as reference for further analysis regarding the effects of the pandemic on the acute stroke care [24] and also for treatment rates among AIS patients with COVID-19 [25].

## Methods

The methods for this analysis have been previously described in detail [2]. Analyses were based upon the statistical evaluation of the German Diagnosis-Related Groups (G-DRG) data from 2019 and 2016 (DRG-statistic, Federal Statistical Office, www.destatis.de), enabling the assessment of the number of ischemic stroke patients based upon the patients’ place of residence.

We extracted all cases with the ICD-10 main diagnosis I63 (acute ischemic stroke). Cases being transferred once or multiple times from one hospital to another either for acute stroke therapy and/or early rehabilitation were censored appropriately to avoid double and multiple coding (exclusion of “discharge key 06”).

The AIS recanalization therapies were categorized using the corresponding Operating and Procedure (OPS) Key for IVT (OPS code 8–020.8) and MT (OPS 8–836.80) in combination with I63 as main diagnosis. In addition, the following OPS codes in combination with the I63 main diagnosis were analyzed to access SUC: 8–981.0 (stroke unit treatment for more than 24 h and less than 72 h); 8–981.1 (stroke unit treatment for more than 72 h); 8-98b.*0/*1 (other acute stroke treatment without / with tele-consultation).

Regional analyses were executed by data aggregation considering the 412 German administrative districts and cities. To avoid bias, we excluded all cases of foreign or unknown place of residence from regional statistics.

For the statistical analyses, we used the data from 2016 and 2019 to assess the differences in treatment rates over the past 3 years. Statistical differences in categorical variables between patients were calculated using chi-squared test (χ^2^) and for continuous variables using t-test. *P* < 0.05 was defined as level of statistical significance.

## Results

**Fig. 1:**
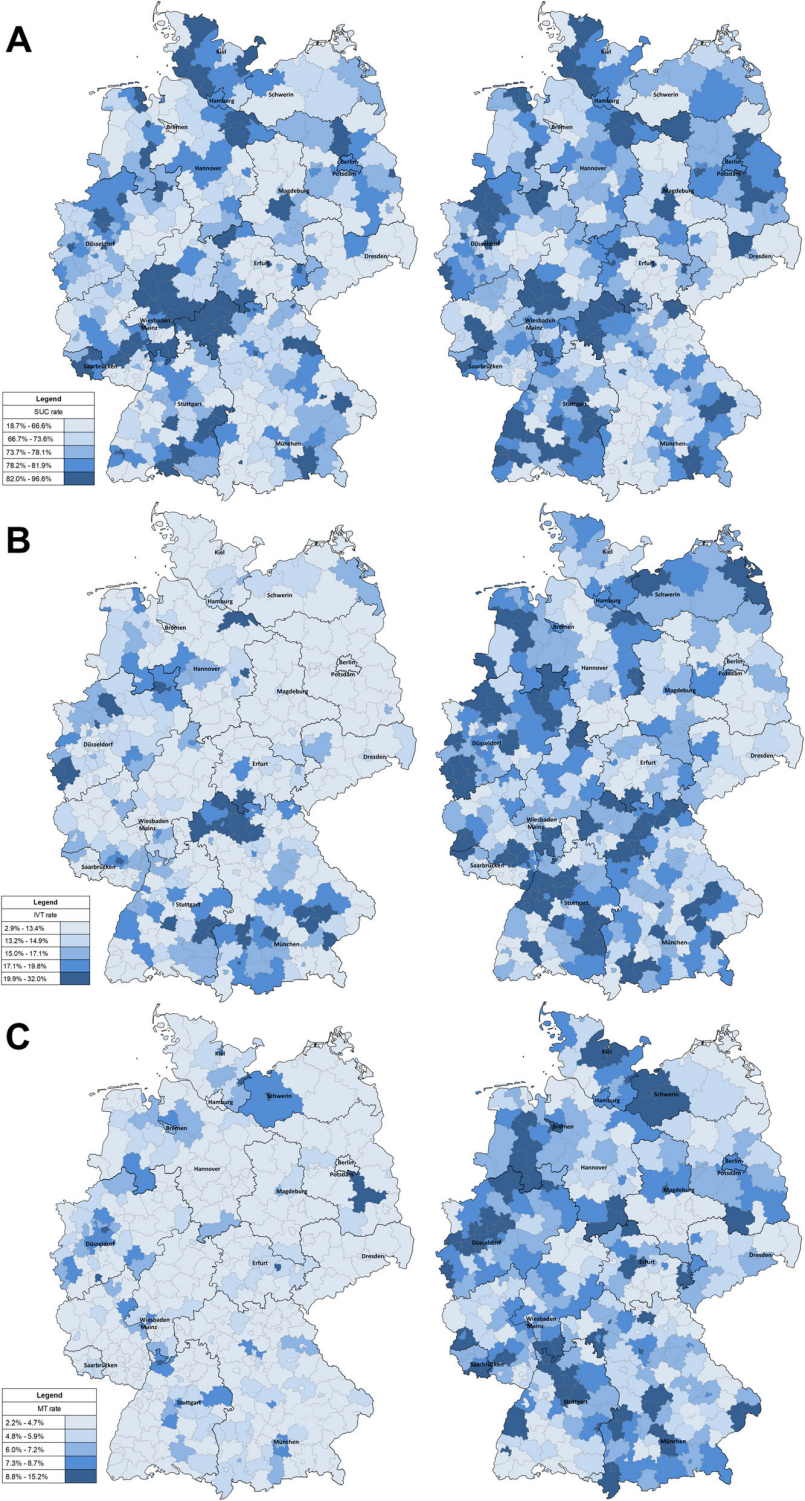
Comparison of the regional distribution of SUC, IVT and MT rates in 2016 and 2019. Regional distribution of SUC (**a**), IVT (**b**) and MT (**c**) rates in 2016 (left) and 2019 (right) for all 412 German districts and cities according to place of patients´ residence. IVT indicates intravenous thrombolysis, MT mechanical thrombectomy, SUC stroke unit care

The administrative hospital data of all acute care hospitals in Germany showed a slight decrease of 1.0% for the total number of hospitalized AIS patients from 2016 (*n* = 227,687) to 2019 (*n* = 225,531). The mean age of patients with ischemic stroke was 74.0 years in 2016 as compared to 74.2 years in 2019. The gender distribution remained unchanged between 2016 and 2019 (Table 1). Less patients with AIS were admitted to SU in 2016 as compared to 2019 (72.1% vs. 74.4%, *p* < 0.001; Table 1). In 2019, the regional distribution of SUC rates was highly variable in Germany, ranging from 18.7 to 96.6% of all AIS patients (Fig. 1a).

**Table 1:** Rates and demographic data for acute ischemic stroke, intravenous thrombolysis, mechanical thrombectomy and stroke unit treatment for the year 2016 and 2019 in Germany

	2016	2019	***p***-Value
Ischemic strokes (ICD 163), n	227,687	225,531	
Age, mean ± SD	74.0 ± 4.8	74.2 ± 4.9	*p* < 0.001
Sex, male, n (%)	118,051 (51.8%)	118,755 (52.7%)	*p* < 0.001
SUC overall, n (%)	164,270 (72,1%)	167,799 (74.4%)	*p* < 0.001
Age, mean ± SD	73.5 ± 4.8	73.6 ± 4.8	*p* < 0.001
Sex, male, n (%)	87,223 (53,1%)	90,108 (53,7%)	*p* < 0.001
SU rate < 80 years, %	74,1%	76,1%	*p* < 0.001
SU rate ≥ 80 years, %	68,8%	72,9%	*p* < 0.001
IVT overall, n (%)	33,916 (14.9%)	36,745 (16.3%)	*p* < 0.001
Age, mean ± SD	73.4 ± 4.7	73.6 ± 4.6	*p* < 0.001
Sex, male, n (%)	17,502 (51,6%)	19,430 (52,9%)	*p* < 0.001
IVT rate < 80 years, %	15.2%	16,7%	*p* < 0.001
IVT rate ≥ 80 years, %	14.4%	15,9%	*p* < 0.001
MT overall, n (%)	9795 (4.3%)	16,135 (7.2%)	*p* < 0.001
Age, mean ± SD	72.6 ± 4.8	74.2 ± 4.9	*p* < 0.001
Sex, male, n (%)	4653 (47,5%)	7332 (45,4%)	*p* = 0.002
MT rate < 80 years, %	4.6%	7.0%	*p* < 0.001
MT rate ≥ 80 years, %	3.8%	7.3%	*p* < 0.001

**Legend:** Percentage values are based on all patients with ICD code I63

*ICD* International Statistical Classification of Diseases and Related Health Problems, *SUC* Stroke unit care, *IVT* Intravenous thrombolysis, *MT* Mechanical thrombectomy, *SD* Standard deviation

The nationwide IVT rate in AIS patients significantly increased from 14.9% in 2016 to 16.3% in 2019 (*p* < 0.001; Table 1). However, there was still a high regional variation in the IVT rate between the 412 different districts in Germany (2016: 2.4 to 28.0%; vs 2019: 2.9 to 32.0%; Fig. 1b). The mean age of patients receiving IVT was 73.4 years in 2016 as compared to 73.6 years in 2019. The IVT rate of patients over 80 years increased from 14.4% in 2016 to 15.9% in 2019. In line with the gender distribution for AIS patients, numerically more male than female AIS patients received IVT (Table 1).

The MT rate increased significantly from 4.3% in 2016 to 7.2% in 2019. There was still a high regional variation between the different districts (2016: 0 to 11.2% vs. 2019: 1.4 to 15.2%, Fig. 1c). Based on patients’ residence, each of the 412 districts coded at least one MT procedure in 2019. The mean age of AIS patients receiving MT treatment was 72.6 years in 2016 and 74.2 years in 2019 (*p* < 0.001, Table 1). In 2016, the MT rate in AIS patients younger than 80 years was higher than in patients ≥80 years (4.6% vs. 3.8%, *p* < 0.001). In 2019, it was the opposite with a higher endovascular treatment rate in AIS patients ≥80 years as compared to AIS patients younger than 80 years (7.3% vs. 7.0%, *p* = 0.007).

Figure 1 depicts the regional distribution of SUC, IVT and MT rates in 2016 and 2019 for all 412 German cities and districts according to place of patients´ residence.

## Discussion

This update of the nationwide analysis of acute ischemic stroke care in Germany reveals almost stable absolute case numbers of patients being hospitalized for AIS between 2016 and 2019. The number of AIS patients receiving at least 24 h of stroke unit treatment has increased significantly to 74.4% which is associated with the general increase of DSG certified Stroke Units from 2016 (*n* = 291) to 2019 (*n* = 330). Up to date, only three quarters of AIS patients receive SUC which should ideally be offered to all stroke patients given the strong evidence for a better long-term functional outcome [26, 27]. More than 50% of the 412 cities/districts report SUC rates above 75%, while only 10 % of the districts reported SUC rates lower than 60%, indicating a broad national availability of this treatment. However, there are still regions, mostly in eastern and southeastern Germany, with low SUC rates, where further structural medical and health policy efforts may are needed to increase SUC rates. Giving the strong guideline recommendations for SUC, we should aim to further increase SUC rate in the future.

Regarding IVT, we documented a further increase in the IVT rate to 16.3% in 2019 with still existing regional variation and lower treatment rates in some parts of Germany. AIS patients from all 412 German cities and counties had access to IVT in 2019. More stroke telemedicine networks like e.g. TEMPiS in Bavaria and SOS-NET in Saxonia might help to further increase the IVT rate and also to earlier identify patients suitable for endovascular treatment. Overall, the IVT rate in Germany was more than twice as high as compared to the European average which was calculated at 7.3% in 2018 [28].

The “WAKE-UP” stroke trial published in May 2018 [18] might have had an impact on the increasing IVT rate observed in 2019. We do expect a further increase of the overall IVT use in the wake of the Extend trial [19], ECASS4 [20] and the subsequent individual patient data meta-analysis [29], given that the incidence of wake-up and unknown time window strokes is estimated to be between 15 and 25% [30].

**Fig. 2:**
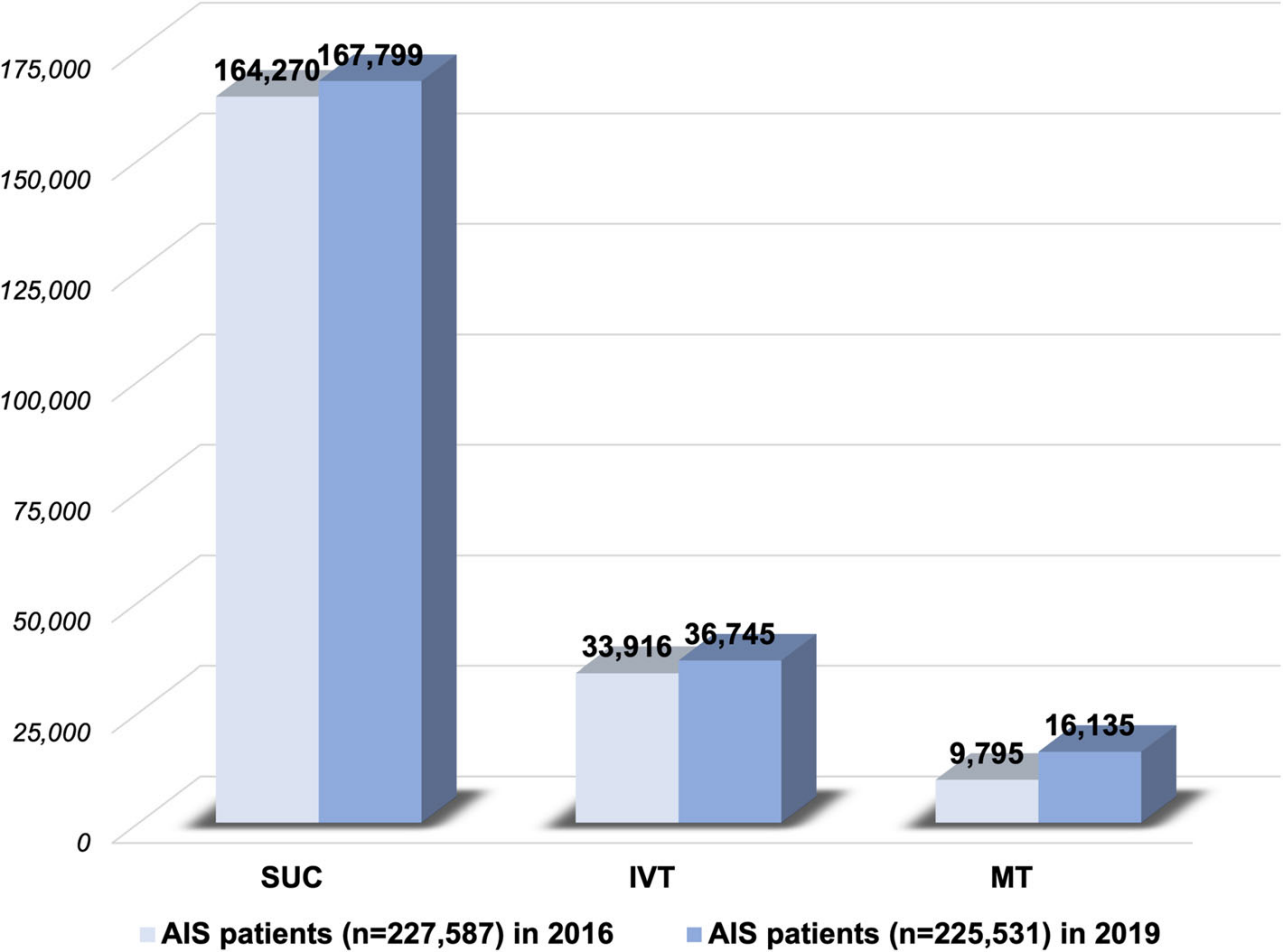
Absolute numbers of SUC, IVT procedures and MT procedures in 2016 and 2019. Bar chart demonstrating the absolute numbers of IVT and MT procedures as well as the absolute numbers for AIS patients treated on stroke units for the years 2016 and 2019. AIS indicated acute ischemic stroke, SUC stroke unit care, IVT intravenous thrombolysis, MT mechanical thrombectomy

With 5 years of evidence after proofing the benefit of MT with stent retrievers in AIS patients with anterior LVO [11], we found an overall MT rate of 7.2% in 2019 which is a strong further increase as compared to the MT rate in 2016 (4.3%). Mechanical thrombectomy is a routine treatment strategy in Germany with over 16,000 treatments in 2019 (Fig. 2). The mean age in AIS patients receiving MT treatment was substantially higher in 2019 as compared to 2016. More AIS patients with an age of ≥80 years received MT. As the overall age of hospitalized AIS patients remained nearly unchanged, this finding underlines the need and use of MT especially in the higher age groups. In general, the MT rate in Germany is continuously rising and also far above the European average, estimated at 1.9% [28]. In 2019, each of the 412 districts/cities in Germany had at least one resident treated with MT indicating an overall availability of this interventional procedure as opposed to 2016 [2]. Nevertheless, there are still major differences in the MT rate in various regions in Germany with lower rates found in the rural eastern and southern parts of the country [31], but these regional differences were more pronounced in 2016 [2]. Just like for IVT treatment strategies, it will be a continuous task to implement MT with an 24/7 availability on the highest quality level, as the indication (e.g. posterior circulation stroke, distal medium vessel occlusion, contraindication to IVT) and the time window between symptom onset and intervention is also growing for MT. [16, 17, 21, 22, 32]

Our data refer to the overall number of patients being treated with the main diagnosis of ischemic stroke (ICD I63), irrespective of, e.g., symptom onset, clinical details or basis of indication, such as vascular occlusion status or symptom severity, which is the major drawback of our study. Furthermore, our analysis on the treatment rates is based on the operating and procedure key coding in the German DRG system which is defined by several structural characteristics, without consideration of other relevant quality factors like procedural treatment times etc. Regarding SUC, the prerequisites for the coding of the therapeutic procedure OPS 8–981 require a special structure of in-hospital service (e.g. monitoring with a minimum duration of 24 h, physiotherapeutics and logopedics as needed). However, a specific certification, like it is given by the DSG, is not mandatory. However, these administrative data have high quality and accuracy because registration of all ischemic stroke cases and acute treatment procedures is a prerequisite to get financial compensation, and the coding of operating and procedure keys for MT, IVT and SUC are closely controlled by medical services of the health insurances. The system itself assures that one procedure refers only to one case, even if the patient has been transferred to a second hospital for MT. Furthermore, no change in coding standards occurred in the analyzed time period. Thus, in contrast to observational regional registries, our data are very robust for Germany with a very low risk of missing patients, double coding of procedures, resulting in high validity and consistency.

## Conclusion

We documented increasing rates of recanalization procedures and SUC in Germany. As there is growing evidence for an extended time window for MT and IVT in a selected number of patients, we assume that the increase in the application of these procedures will continue in the future. It is an ongoing and challenging task to provide these interventional therapies for each eligible AIS patient in Germany.

## Data Availability

Data according to §21 KHEntgG and §24 para. 2 KHG; official data on file, source: Destatis, www.destatis.de.

## Abbreviations

AIS: Acute ischemic stroke
COVID-19: Coronavirus disease 2019
G-DRG: German Diagnosis-Related Groups
IVT: Intravenous thrombolysis
LVO: Large vessel occlusion
MT: Mechanical thrombectomy
OPS: Operating and Procedure Key
rt-PA: Recombinant tissue plasminogen activator
SUC: Stroke Unit care (AIS)
